# X-Ray Diagnosis of Hair Ball in Stomach

**Published:** 1914-01

**Authors:** 


X-Ray Diagnosis of Hair Ball in Stomach. Albert Ramsbottom and Alfred E. Barclay of Manchester, Eng., *Med. Chron.*, Oct., 1913, describe a case at first considered splenic tumor, the blood showing, however, 65% haemoglobin, 4,800,000 reds, 5,760 whites and no myelocytes. The diagnosis was made by combined fluoroscopy and palpation. The bismuth flowed to the left of the tumor, showing that, as the greater curvature was not displaced, the tumor could not be the spleen. By manipulation, it was shown that the tumor which gave no shadow itself, was within the stomach, and that it was freely movable. The diagnosis of hair ball was, therefore, made in spite of denial of trichophagy and the diagnosis was verified by a successful gastrotomy. Later, the patient's brother stated that she had eaten all her hair during convalescence from scarlet fever, 20 years previously. "One of us" had suggested the same diagnosis on inspecting a radiograph of a case not directly under observation and operation has corroborated the diagnosis (*Arch. of the Röntgen Ray,* July, 1913.) The present case is of interest as being the first positive diagnosis of hair ball made in advance and also with reference to the discussion as to the relative value of radiography and fluoroscopy.

